# Translation and validation of Warmometer, a tool for assessing warmth in patient-provider relationships, for use in Brazilian Portuguese

**DOI:** 10.1590/1516-3180.2018.0035160318

**Published:** 2018-03-29

**Authors:** Marieta Sodré Brooke, Mary Uchiyama Nakamura, Jorge Kioshi Hosomi, Meireluci Costa Ribeiro, Nelson Sass

**Affiliations:** I MD. Postgraduate Student, Department of Obstetrics, Escola Paulista de Medicina (EPM), Universidade Federal de Sao Paulo (Unifesp), São Paulo (SP), Brazil.; II MD, PhD. Professor, Department of Obstetrics, Escola Paulista de Medicina (EPM) Universidade Federal de Sao Paulo (Unifesp), São Paulo (SP), Brazil.; III MD, PhD, Researcher, Department of Obstetrics, Escola Paulista de Medicina (EPM), Universidade Federal de Sao Paulo (Unifesp), São Paulo (SP), Brazil.; IV PhD. Licensed Clinical Social Worker and Researcher, Department of Obstetrics, Escola Paulista de Medicina (EPM), Universidade Federal de Sao Paulo (Unifesp), São Paulo (SP), Brazil.; V MD, PhD. Professor, Department of Obstetrics, Escola Paulista de Medicina (EPM), Universidade Federal de Sao Paulo (Unifesp), São Paulo (SP), Brazil.

**Keywords:** Empathy, Physician-patient relations, Survey and questionnaires, Psychometrics

## Abstract

**BACKGROUND:**

Empathy in the patient-provider relationship is associated with important outcomes in healthcare practice. Our aim was to translate and validate Warmometer, a visual tool for assessing warmth in patient-provider relationships, for use in Brazilian Portuguese.

**DESIGN AND SETTING:**

Cross-sectional study conducted at an antenatal clinic of a public university hospital in São Paulo, Brazil.

**METHODS:**

The instrument was translated into Brazilian Portuguese and culturally adapted. It was tested for reliability and validity among 32 pregnant women, between June 2015 and January 2016. To assess construct validity, it was correlated with the Consultation and Relational Empathy (CARE) scale (gold standard for patient-provider relationships) and the Interpersonal Reactivity Index (IRI).

**RESULTS:**

The translated version of Warmometer had good face and content validity, low intra-observer reproducibility (intraclass correlation coefficient, ICC: 0.224; 95% confidence interval, CI -0.589 to 0.621;P = 0.242) and high inter-observer reproducibility (ICC: 0.952; 95% CI 0.902 to 0.977; P < 0.001). There was a strong correlation between Warmometer and CARE (*r =* 0.632) and a weak correlation between Warmometer and IRI (*r* = 0.105).

**CONCLUSIONS:**

Warmometer was translated, culturally adapted and validated for use in Brazilian Portuguese. The translated version is a reliable tool for assessing the degree of empathy perceived by the patient in a patient-provider relationship.

## INTRODUCTION

Perceived warmth or empathy is defined as a social-emotional ability with affective and cognitive components. These components refer to the ability to share and understand the emotions of others, respectively.^1^ Empathy in the patient-provider relationship is associated with important outcomes such as higher patient satisfaction^2^ and adherence to treatment,^3^ as well as with increased diagnostic accuracy^4^ and positive health outcomes.^5^^,^^6^ During pregnancy, empathy in the patient-provider relationship is associated with satisfaction with delivery.^7^

There are several questionnaires for evaluating empathy in the patient-provider relationship,^8^^,^^9^^,^^10^ both from the physician’s and from the patient’s perspective. The Interpersonal Reactivity Index (IRI)^11^ and the Consultation and Relational Empathy (CARE) scale^12^ are among the ones most used. The IRI is a first-person tool that allows physicians to evaluate themselves, and it was translated into Brazilian Portuguese by Sampaio et al. in 2011.^13^ The CARE instrument assesses the patient’s perception of empathy in the patient-provider relationship and was translated into Brazilian Portuguese in 2014.^14^

However, written questionnaires can sometimes be difficult to understand and use.^15^ Visual tools not only dispense with the need for in-depth reading and writing skills but also spare the participants from many of the barriers associated with completing written questionnaires.

Warmometer is a tool for measuring the warmth of the patient-provider relationship. It is a self-reporting visual and cognitive tool for assessing the socio-emotional quality of healthcare providers from the patient’s perspective.^8^ This tool was created by Neumann et al. in 2011, in a German hospital specializing in holistic and anthroposophic medicine.

Our objective was to translate, cross-culturally adapt and validate Warmometer for use in Brazilian Portuguese.

## METHODS

### Study design, setting and ethics

This study was approved by the Ethics Committee of the Federal University of São Paulo (CAAE: 20537514.6.0000.5505). All participants signed written informed consent.

First, we translated a 17-item probing questionnaire^8^ created to ensure comprehension of Warmometer (Table 1), here referred to as the “probing questionnaire”. Then, we translated Warmometer itself. It was tested for reliability and validity among participants who were pregnant women, between June 2015 and January 2016, according to the procedures described below. To assess construct validity, it was correlated with the Consultation and Relational Empathy (CARE) scale (gold standard for patient-provider relationships) and the Interpersonal Reactivity Index (IRI).

**Table 1:** Probing cognitive interview questionnaire^8^

	Probe questions
1	What characteristics and type of behavior demonstrated by your physician is your assessment based on? (see Figure 1, presenting an illustration of the Warmometer)
2	How did you arrive at your answer on the thermometer?
3	How do you perceive the way in which the physician talks to you?
4	What do the gestures of your physician mean to you (e.g. whether your physician shakes your hand to welcome you)?
5	Has your assessment of your physician changed since your first contact with him/her?
6	What do you think about the thermometer as a response format? Would you change anything?
7	Can you please repeat the first question in your own words? What is this question about, in your view?
8	Was the question easy for you to understand? Would you change anything?
9	Please indicate how much warmth your ideal physician would show towards you by placing an ‘‘X’’ directly on the thermometer to the left.
10	What characteristics and type of behavior of an ideal physician is your answer based on?
11	Please imagine a person from your personal environment (e.g. family, friends, neighbors or colleagues) who shows great warmth towards you. Indicate how much warmth this person shows to you by placing an ‘‘X’’ directly on the thermometer to the left.
12	What characteristics and type of behavior is your assessment based on?
13	Please imagine a person from your personal environment (e.g. family, friends, neighbors or colleagues) who shows an average amount of warmth towards you. Indicate how much warmth this person shows you by placing an ‘‘X’’ directly on the thermometer to the left.
14	What characteristics and type of behavior is your assessment based on?
15	Please imagine a person from your personal environment (e.g. family, friends, neighbors or colleagues) who shows coldness towards you. Indicate how much coldness this person shows you by placing an ‘‘X’’ directly on the thermometer to the left.
16	What characteristics and type of behavior is your assessment based on?
17	Do you have any other comments on the issue of human warmth in the patient-provider relationship? Or, is there anything else I should know?

### Participants

For this study, healthy women of any gestational age, who were being managed at the antenatal care clinic of a large public university hospital in the city of São Paulo, Brazil, were recruited between June 2015 and January 2016. Participation was voluntary, and the women had to be at least 18 years of age and be able to speak and read Portuguese fluently. Those with psychiatric diagnoses (e.g. dementia or schizophrenia) were excluded.

Firstly, the principal investigator (MB, an obstetrician) approached the physicians working at the clinic to explain the study and invite them to participate. They were informed that they would be asked to fill in a questionnaire (the IRI), immediately after conducting a routine antenatal consultation with each participating woman.

The investigator then approached the women immediately after these consultations and told them about the study. Those who fulfilled the selection criteria and agreed to participate received three written questionnaires (a sociodemographic data collection form, a probing cognitive questionnaire and the CARE measurement tool) and also the Warmometer tool, to be answered individually and anonymously in a private room (first interview). The physician who had just examined the participant also received an IRI form to be filled out individually and anonymously. The completed questionnaires were returned to the investigator and were placed in an opaque envelope marked with the participant’s initials.

Two to three weeks later, at the participant’s next scheduled routine antenatal care visit, another investigator (SO, a psychologist) approached the same participants and asked them to again fill out the probing cognitive questionnaire and to respond to the Warmometer tool, individually, in a private room (second interview). The completed questionnaires were placed into each participant’s opaque envelope. As part of their routine antenatal care, all women in this antenatal clinic are cared for by a multidisciplinary team (obstetrician, psychologist, nutritionist, dermatologist, physiotherapist and nurses) and participate in several additional activities (e.g. exercise sessions, group discussions, hydrotherapy, psychotherapy and massage). Since the women remain in the clinic for several hours, the principal investigator (MB) approached them on the same day, two hours later, and repeated the procedures (third interview) that had been used by investigator 2 (SO). None of the participants received care from the investigators, at any of their antenatal appointments.

The participants’ responses to Warmometer were placed in their individual envelopes. At the end of the study, each of the participants’ envelopes contained 3 Warmometer questionnaires; 1 probing cognitive questionnaire with 17 answers (first interview), 2 probing cognitive questionnaires with 4 answers (to questions 9, 11, 13 and 15) obtained in the second and third interviews, the physician IRI questionnaire obtained in the first interview and the sociodemographic data from each participant.

### Details of the questionnaires

#### The probing cognitive questionnaire

Based on Tourangeau’s model for a cognitive interview questionnaire,^16^ Neumann et al.^8^ developed a 17-item probing questionnaire to ensure comprehension of Warmometer (Table 1). The original questionnaire used descriptive answers to assess four key points:

1. comprehension of a question;
2. retrieval of information from autobiographical memory;
3. use of heuristic and decision-making processes to estimate an answer; and
4. formulation of a response.

Item 2 was not included in the present study because the participants gave responses to the Warmometer tool immediately after their appointment with the physician, and issues with retrieval of information were thought to be very unlikely.

In the first interview, all participants were asked to answer the full probing cognitive questionnaire before giving responses to the Warmometer tool. In the second and third interviews, the women answered only four of the 17 questions of the probing cognitive questionnaire: degree of warmth of the ideal physician (9); degree of warmth of very warm people (11); degree of warmth of averagely warm people (13); and degree of warmth of cold people (15).

#### Warmometer

**Figure 1:**
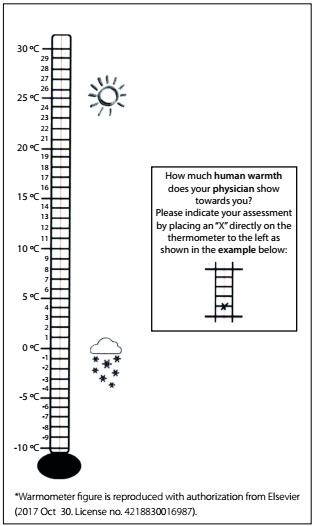
Final version of the Warmometer, as developed by Neumann et al. (2011)^8^*

Warmometer provides a short self-reported assessment by patients of physicians’ warmth, visually represented by a thermometer. The tool was developed based on the concepts of warmth in human relationships. Neumann et al.^8^ considered warmth to be “a higher temperature, a still pleasant feeling, that is no longer cold and not yet hot” and coldness to be the “absence of warmth”. To capture differences in individual perceptions of and preferences for warmth and coldness, these authors created an image of a thermometer with a temperature range from -10 °C to +30 °C (Figure 1). They described a “cold patient-provider relationship” as one that was between 15 °C and 18 °C and a “warm patient-provider relationship” as one that was between 22 °C and 24 °C.^8^

### Steps in the cultural adaptation process

#### The probing cognitive questionnaire

The original version of the probing cognitive questionnaire (Table 1) was translated from English into Brazilian Portuguese by two native Brazilians who were English teachers. This translation was discussed by the two teachers and the main investigator (MB) until consensus was reached.

This initial version was tested on 20 pregnant women who were receiving antenatal care in the study clinic. The questions for which more than 15% of the responses consisted of the option “not applicable” were reviewed and modified. This process produced a second version of the probing cognitive questionnaire, which was tested again on the same 20 participants, on another occasion. This version was considered appropriate if less than 15% of the responses to the questions consisted of the option “not applicable”.

#### Warmometer

**Figure 2:**
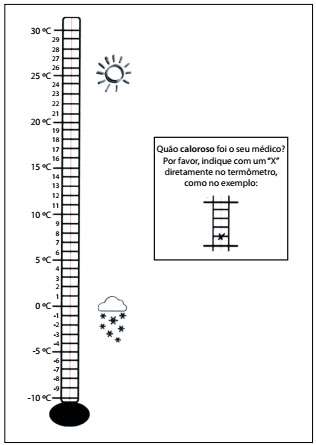
Final version of the Warmometer: translated, culturally adapted and validated for use in Brazilian Portuguese.

We obtained authorization from the main author of Warmo-meter (M. Neumann) to translate the instrument into Brazilian Portuguese. The original version of the instrument was translated from English into Brazilian Portuguese in accordance with the methods recommended for translation and cultural adaptation of health-related quality-of-life and self-reporting measurements.^17^^,^^18^ Two native Brazilians who were English teachers translated the original text (“How much warmth does your physician show towards you?”) independently. These two versions were discussed by the teachers and the main investigator (MS) until a consensus was reached. The new version was translated back into English by two other Brazilian English teachers and this version was compared with the original English text. The Brazilian Portuguese translation of the Warmometer (Figure 2) was tested on a small group of 20 women to identify any problems of comprehension. The investigators assessed whether the consensus version of the translated Warmometer was appropriately adapted to the linguistic and cultural context of the women who would use the instrument, and whether it maintained all the essential characteristics of the original version.

### Assessment of psychometric properties

After translation and cultural adaptation, the final version of Warmometer was tested for reliability and for face, content and construct validity, as detailed below.

Reliability was examined through test-retest procedures in three interviews involving the same participants. In the first interview, 32 participants filled out Warmometer responses. Two to three weeks later, the same participants were approached by two independent investigators at different times (two hours apart) on the same day and were asked to fill out Warmometer responses. We calculated the inter- and intra-test reliability using intraclass correlation coefficients (ICC), with 95% confidence intervals (CI), comparing the individual scores given by the participants in each of the three interviews.

Face validity was determined by reaching a consensus among the investigators involved in translation of the instrument. They evaluated whether the Brazilian version of the Warmometer appeared to measure what it intended to measure.

Content validity refers to how well a test measures the behavior for which it is intended. This needs to be established using a defined standard to compare content or results.^19^ Content validity in this study was evaluated by means of checking the answers that were given in the probing cognitive questionnaire that was used to test the participants’ comprehension of Warmometer and observed whether the participants had any doubts or queries about answering the questions or any suggestions for changes to the questions.

Construct validity refers to the extent to which the new tool conforms to previous ideas or hypotheses about the concepts (constructs) that are being measured.^19^ This was tested by comparing Warmometer with the Consultation and Relational Empathy (CARE) measurement.^12^ CARE is a 10-item self-reporting tool for measuring patients’ perceptions of relational empathy in consultations, which are evaluated on a Likert scale, ranging from 1 (“poor”) to 5 (“excellent”). Higher scores indicate higher levels of empathy. This tool was translated into Brazilian Portuguese by Scarpellini et al.^14^ and has good internal consistency (Cronbach’s alpha of 0.867).

The Interpersonal Reactivity Index (IRI),^11^ which was filled out by the physicians, was also used to test the construct validity of Warmometer. The IRI is a 28-item self-reporting self-evaluation questionnaire consisting of four seven-item subscales, each of which assesses a specific aspect of empathy: perspective taking (PT) scale; fantasy (FS) scale, including three items of the fantasy-empathy (F-E) scale;^20^ empathic concern (EC) scale; and personal distress (PD) scale. Each of these subscales is composed of seven propositions, which are graded by the respondents using a Likert scale, ranging from 1 (“does not describe me well”) to 5 (“describes me very well”). Higher scores indicate higher levels in each of these dimensions, and the sum of the scores of all subscales is used to calculate the overall level of empathy. The IRI was translated into Brazilian Portuguese by Sampaio et al. in 2011 and has good internal consistency (Cronbach’s alpha of 0.861).^13^ We used Pearson correlation coefficients to assess the association between the Warmometer scores and the CARE and IRI scores. R values < 0.30, from 0.30 to 0.50 and > 0.50 were interpreted as indicative of weak, moderate and strong correlations, respectively.^21^ P*-*values < 0.05 were considered statistically significant.

We used the Statistical Package for the Social Sciences (SPSS) software, version 21 (IBM Corporation, Armonk, NY, USA), for the statistical analyses.

## RESULTS

We recruited 20 pregnant women for the cultural adaptation of the translations of the probing cognitive questionnaire and Warmometer, and all of the recruited women agreed to participate. The translated version of Warmometer was well understood by all participants. However, over 15% of the women did not understand question 1 of the probing cognitive questionnaire. The translation of this question was then modified, and the revised version of the probing questionnaire was tested again on the same 20 pregnant women. Seventeen women considered that this second version was appropriate.

A total of 40 pregnant women agreed to participate in the validation phase (including the previous 20). Eight were excluded because they returned incomplete questionnaires. Initially, we told all the participants about the importance of the study and that all fields of the questionnaires should be answered. However, to ensure their comfort and confidentiality, the participants were left alone while answering the questionnaires. When we performed the statistical analysis, we needed to compare the responses to Warmometer with the answers given to the other instruments (the 17 items of the probing questionnaire, the Consultation and Relational Empathy (CARE) scale and the IRI. At this stage, we had to exclude 8 women because they did not completely fill in all of these instruments. Thus, a total of 32 women were included in the final analyses. Their mean age was 30.0 years (standard deviation ± 4.8), ranging from 20 to 41 years. Nearly half of them (47%; n = 15) had < 9 years of formal education; 19% (n = 6) had 9-12 years; and 34% (n = 11) had > 12 years. Most of them (78%; n = 25) were white; 1.2% (n = 4) were black; and 0.9% (n = 3) were of mixed color. Five physicians were invited to participate in the study and filled out IRI questionnaires.

These 32 participants provided the following responses during the first interview (the probing cognitive questionnaire with 17 questions):

- 30 (94%) stated that they would not change anything in the format of the instrument and considered the questions to be “easy to understand”;
- 24 (75%) responded that their assessment was based on the “attention” that they received from the physician;

The following attitudes and behaviors were mentioned by the women as examples of warmth:

- Warmth from their attending physician: attention, tone of voice, eye contact, greeting, smiling and introducing himself/herself;
- Warmth from an ideal physician: being available, calm, happy to be in the consultation, eye contact, showing interest and caring for the patient.

Table 2 presents the mean physician warmth scores (temperatures) according to the participants’ responses to the Warmometer question: “*How much warmth does your physician show towards you?”* and their answers to questions 9, 11, 13 and 15 (the ones that were responded in the three interviews and are part of Warmometer) of the probing cognitive questionnaire, in the three interviews.

**Table 2:** Physician warmth temperatures perceived by 32 pregnant women and responses to probe questions 9, 11, 13 and 15 (see Table 1)

	Mean temperature		
	1^st^ interview^a^	2^nd^ interview^b^	3^rd^ interview^c^
Physician warmth	25.5 ± 5.6	25.0 ± 5.9	25.4 ± 5.5
Ideal physician warmth	27.3 ± 3.8	26.4 ± 3.8	25.6 ± 4.5
Person of great warmth	28.1 ± 3.2	25.8 ± 4.1	26.5 ± 3.9
Person of average warmth	18.8 ± 6.6	16.9 ± 6.8	16.8 ± 6.8
Cold person	3.8 ± 5.7	7.9 ± 8.3	6.7 ± 7.2

^a,b^conducted by interviewer 1; ^c^conducted by interviewer 2. All values expressed as mean ± standard deviation.

To evaluate test-retest reliability, a total of 32 participants completed the Brazilian Portuguese version of Warmometer three times. The women took an average of 2-3 minutes to answer the questions. There was no significant intra-observer reproducibility, based on the responses obtained by the principal investigator in the first interview and in the second interview 2-3 weeks later, (P = 0.491; Pearson correlation coefficient, *r* = 0.126). However, there was significant inter-observer reproducibility, based on correlation of the responses between the second and third interviews (conducted two hours apart by different investigators), (P < 0.001, *r* = 0.912).

Homogeneity analysis, using ICC, showed weak intra-observer correlation without statistical significance (ICC: 0.224; 95% confidence interval, CI -0.589 to 0.621; P = 0.242) and strong, statistically significant inter-observer correlation (ICC: 0.952; 95% CI 0.902 to 0.977; P < 0.001) (Table 3).

**Table 3:** Intra and inter-observer reliability of Warmometer, as assessed in a sample of 32 pregnant women

Warmometer Temperatures	Intraclass correlation			Interclass correlation		
	ICC	95% CI	P-value	ICC	95% CI	P-value
Physician warmth	0.224	-0.589-0.621	0.242	0.952	0.902-0.977	< 0.001
Ideal physician warmth	0.584	0.147-0.797	0.009	0.856	0.705-0.930	< 0.001
Person with great warmth	0.424	-0.180-0.719	0.065	0.908	0.812-0.955	< 0.001
Person of average warmth	-0.136	-1.328-0.445	0.638	0.777	0.543-0.891	< 0.001
Cold person	0.205	-0.629-0.612	0.263	0.727	0.440-0.866	< 0.001

ICC = intraclass correlation coefficient; CI = confidence interval.

### Validity

Almost all participants (94%) stated that Warmometer was easy to understand during the probing questionnaire evaluation, and that they would not change anything in its format or questions. Based on this response, the multidisciplinary team established the face and content validity of the Brazilian Portuguese version of Warmometer.

Construct validity was determined by comparing the Warmometer scores with the CARE and IRI scores in a sample of 32 pregnant women. There was a strong, statistically significant correlation between the Warmometer and CARE scores (*r* = 0.632; P < 0.001). There was a weak, statistically insignificant correlation between the Warmometer and IRI scores (*r* = 0.105; P = 0.567).

## DISCUSSION

The temperature ratings from Warmometer that our participants gave and their responses to the probing cognitive questionnaire seem to confirm the close relationship between warmth, empathy and social relations. The average temperature ratings given by our participants to their attending physician, in the three interviews, were approximately 25 °C, and this was also very close to the ideal temperature rating for physicians that they gave. This means that they felt welcomed by the attending physician and that the consultations were within their expectations.

Several studies have shown that empathy, or perceived warmth in the patient-provider relationship, is associated with positive health outcomes.^2^^,^^3^^,^^4^^,^^5^^,^^6^^,^^22^ This is especially important during pregnancy, a special period in a woman’s life, when a good relationship between the patient and her healthcare providers can promote satisfaction and contribute towards creating good memories of the birth experience.^7^ In 2006, Domingues, Santos and Leal^7^ conducted a cross-sectional study in a public hospital in Rio de Janeiro, Brazil, to assess the opinions and feelings of 250 women about the care received from healthcare professionals. Nearly 75% of the women (139/187) with a positive perception of their healthcare team reported their delivery experience as good/very good, compared with only 44% (26/59) of those who had a negative perception of their healthcare professionals (P < 0.001).

Our pregnant participants stated that they took into consideration not only what the physician said but also the way in which he/she spoke. “Attention”, “tone of voice” and “eye contact” were mentioned by many of our participants as characteristics of warmth in the patient-provider relationship, thus indicating that patients are highly sensitive to nonverbal communication and that this is important to them. According to physiology studies, nonverbal communication is detected more rapidly by the brain (in the amygdala) than is verbal content (in the prefrontal cortex).^23^

In our study, 32 healthy pregnant women gave responses to Warmometer on three different occasions, whereas 16 individuals (8 patients and 8 healthy volunteers) were involved in the development of the original instrument.^8^ Our low and statistically insignificant intra-observer ICC score may have been due to the treatment that these women could receive prior to responding to the questionnaire in the clinic (e.g. massage, physiotherapy, psychotherapy or hydrotherapy). In contrast, the inter-observer ICC scores (0.902 to 0.977) and the total ICC score (0.952) were high and statistically significant (P < 0.001). This is an interesting finding, since Warmometer was applied by professionals with different backgrounds (an obstetrician and a psychologist), which suggests that the instrument can be used by different types of healthcare professionals.

There was a good correlation between the Brazilian Portuguese Warmometer and the CARE measurement, which is considered to be the gold standard for measuring empathy in patient-provider relationship. However, the CARE questionnaire does so in written form, not visually as in Warmometer. On the other hand, the correlation between the Brazilian Portuguese Warmometer and IRI was not statistically significant (P = 0.187). This may have been because Warmometer and IRI assess two different points of view about the empathy of the relationship: respectively, the patient’s and the physician’s perceptions of warmth. In other words, not all doctors who consider themselves empathic are perceived by patients as being warm. However, contrary to our findings, a Canadian study involving 70 nurses and 70 patients in acute care settings^24^ reported that there was a positive correlation between the measurements of nurse-expressed empathy and patient-perceived empathy.

Although empathy receives little attention during medical training or clinical practice, several studies have shown that not only competence but also empathy is critical to improving health outcomes.^2^^,^^3^^,^^4^^,^^5^^,^^6^^,^^7^^,^^24^ Even though the instrument was not developed specifically for pregnant women, we decided to validate the instrument in this population because we know the importance (both from a theoretical and a practical perspective) of empathy in the patient-provider relationship during pregnancy. This is a special period in a woman’s life during which a good relationship between her and the healthcare providers can promote satisfaction and contribute towards creating good memories of the birth experience.^7^

One strong point of this study is that, to the best of our knowledge, this is the first translation of Warmometer to another language. One limitation was that all participants were pregnant women. Therefore, our findings need to be confirmed in future studies involving different populations.

## CONCLUSION

Warmometer was translated, culturally adapted and validated for use in Brazilian Portuguese. This version of the tool has good reliability and validity, and it can be used to assess Brazilian patients’ perceptions of warmth among their healthcare providers.
